# Selective Anisotropy of Mechanical Properties in Inconel718 Alloy

**DOI:** 10.3390/ma14143869

**Published:** 2021-07-11

**Authors:** Yu Liang, Jun Ma, Baogang Zhou, Wei Li

**Affiliations:** 1 College of Materials and Metallurgy, Guizhou University, Guiyang 550025, China; junma19930521@163.com (J.M.); zbg1140466928zbg@163.com (B.Z.); wli1@gzu.edu.cn (W.L.); 2 Guizhou Key Laboratory for Mechanical Behavior and Microstructure of Materials, Guizhou University, Guiyang 550025, China; 3 National & Local Joint Engineering Laboratory for High-Performance Metal Structure Material and Advanced Manufacturing Technology, Guiyang 550025, China

**Keywords:** Inconel718 alloy, mechanical properties, anisotropy, delta phase

## Abstract

Mechanical anisotropy behaviors are investigated in slightly rolled Inconel718 alloy with string-like δ phase and carbides produced during various solid-solution and aging treatments. A weak anisotropy in the strengths and rupture properties at 650 °C is visible, whereas ductility, i.e., reduction in area (RA) and impact toughness (CVN), presents a sound anisotropy behavior. MC carbides promote the operation of slip systems and thus are conducive to weakening the strength anisotropy. The RA anisotropy mainly stems from high-density δ phase particles that provide more crack nucleation sites and stimulate rapid propagation because of the shorter bridge distance between micro-cracks at the rolling direction. In contrast, CVN anisotropy arises from both δ phase and carbides at a lower solid-solution temperature of 940 °C but only depends on carbides at 980 °C where the δ phase fully dissolves. Apart from dislocation motions operated at room temperature, the activated grain boundary processes are responsible for the weak anisotropy of rupture properties at the elevated temperature. This work provides a guideline for technological applications in the hot working processes for Inconel718 alloys.

## 1. Introduction

Inconel718 (IN718) alloy is widely used in aerospace engines, industrial gas turbines, high-temperature ring structures, nuclear industrial components and fasteners because of its good mechanical properties, corrosion resistance, welding performance and hot workability [1,2,3]. It consists of γ-matrix with γ″-Ni3Nb and γ′-Ni3 (Al, Ti) precipitates, whereas the former triggers a remarkable hardening effect and the latter one only brings a slight additional strength to the alloy [4,5,6]. Thus, solid-solution and aging treatments are recommended to improve the mechanical properties [7]. Moreover, a small quantity of δ-Ni3Nb phase and MC carbides are visible. However, they may play a significant role in mechanical properties. For example, the δ phase located at grain boundaries (GB) stimulates crack propagation, whereas such GB δ phase with a proper volume improves the creep properties of the alloy [8]. Furthermore, the δ phase exhibits a preferred orientation and forms a string during forging, which triggers the anisotropic deformation behavior [9]. Crack propagates more slowly in the perpendicular direction to the string than that in the parallel direction. This is attributed to the string arrangement of the δ phase [10]. On the contrary, it is the arrangement of carbides that accounts for the anisotropy of mechanical properties at room temperature, and the δ phase only accelerates the crack growth, reported by Teimouri [11]. It is further reported [12] that the propagation direction is dependent on the arrangement of carbides in coarse-grained materials during creep testing. Thus, the two particles play a confusing role in mechanical properties, i.e., the anisotropy, which is still not well understood.

Herein, such kinds of particles aligned along the rolling direction are tailored via a slight rolling deformation followed by various solid-solution and aging treatments in the IN718 alloy, where the δ phase is gradually dissolved at a higher solid-solution temperature but without dissolution of MC carbides. The insignificant texture is developed because of slight deformation. The effect of δ phase and MC carbides on the anisotropy of mechanical properties is systematically investigated in this material. The results can provide guidelines to manufacture high-performance IN718 alloy.

## 2. Materials and Methods

**Figure 1:**
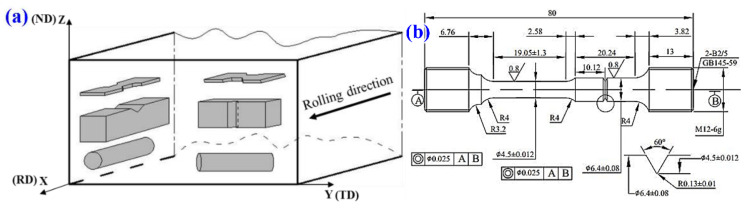
(**a**) Schematic of the machined specimens and their orientation in the billet; (**b**) Specimen dimensions used for stress rupture tests (unit: mm).

An Inconel 718 bar with a section dimension of 40 mm × 90 mm was used in the present work, whose composition is Ni-18.77 Cr-18.05 Fe-5.22 Nb- 3.2Mo-1.09 Ti-0.55 Al-0.03 C (wt.%). The hot-rolled materials were subjected to a solid-solution treatment at 940–980 °C for 1 h, followed by water cooling to obtain different amounts of δ phase, and then doubly-aged at 720 °C for 8 h and 620 °C for 8 h to tailor the strengthening γ″ phase. The coordinate system, including the rolling direction (RD), transverse direction (TD), and normal direction (ND), is established in the hot-rolled bar, as shown in Figure 1a. Samples for all experiments are machined to have two different orientations (Figure 1a) labeled as X (parallel to the X-axis) and Y, respectively. Tensile tests were performed on an MTS810 testing machine at ambient temperature with a tensile rate of 1 mm/min, and the gauge dimension is 10 mm × 3 mm × 35 mm. Charpy V-notched impact samples with a dimension of 10 × 10 × 55 mm^3^ were tested at room temperature on the MTS ZBC2302-4 Pendulum Oscillometric impact tester with a speed of ~5.24 m/s. The stress rupture tests were carried out at 650 °C for 250 h under 690 MPa, followed by a continuous stress increase of 34.5 MPa at intervals of 8 h until failure. The rupture time and ductility, i.e., elongation (EL) and reduction in area (RA), were measured after testing. The specimen dimensions used for rupture tests are shown in (Figure 1b). All samples with orientations X and Y are tested three times, and hereafter designated as T-RD and T-TD, respectively. For example, the 940-RD sample represents a sample solid-solution treated at 940 °C and tested at RD (or had the orientation X).

The microstructure was examined under the optical microscope (OM, Leica DMI5000M) and scanning electron microscope (SEM, SUPRA40) after electrolytic etching in ~10% oxalic acid solution with a direct current of 0.05 A for 30–60 s. Energy dispersive spectroscopy (EDS) was used to determine the chemical composition of particles. After mechanically grinding, samples for transmission electron microscopy (TEM) observation were electropolished in an alcohol solution of 5% HClO4 at −20 °C and 20 V. The X-ray diffraction (XRD) patterns were obtained using a GNR Explorer diffractometer with Cu Kα radiation at 40 kV and 30 mA with a scanning speed of 2 °/min. After tensile, impact and rupture testings, the surface morphology was also observed under SEM (SUPRA40).

## 3. Results

### 3.1. Microstructure

**Figure 2:**
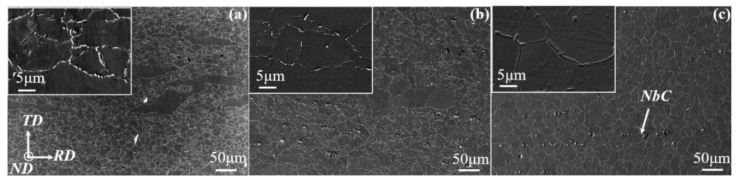
Microstructural morphology on TD–RD plane of Inconel 718 alloy treated at (**a**) 940 °C, (**b**) 960 °C and (**c**) 980 °C.

Figure 2 shows microstructural morphologies on the RD–TD plane of samples subjected to solid-solution and aging treatments. Slightly elongated grains with a size of ~20 μm are visible for the sample treated at a solid-solution temperature of 940 °C (Figure 2a). A string of particles decorating the grain boundary is determined to be δ phase by EDS analysis (not shown here). These particles are gradually dissolved and disappeared at a higher treated temperature, accompanied by the grain growth and the formation of equiaxed grains (Figure 2b,c).

**Figure 3:**
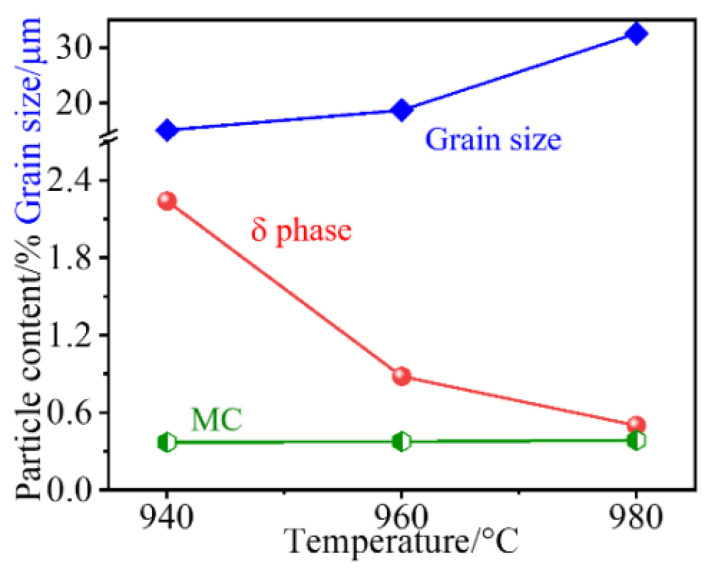
Grain size and fractions of particles (δ phase and MC carbides) on TD–RD plane at various solid-solution temperatures.

Besides, particles with bigger sizes are distributed along the RD and identified as MC carbides (e.g., NbC). These carbides are almost unchanged at all treated temperatures, as supported by the statistical results in Figure 3. There is an opposite trend between grain size and the amount of δ phase. This indicates that grain coarsening mainly stems from the dissolution of the δ phase that strongly pins the grain boundary migration.

**Figure 4:**
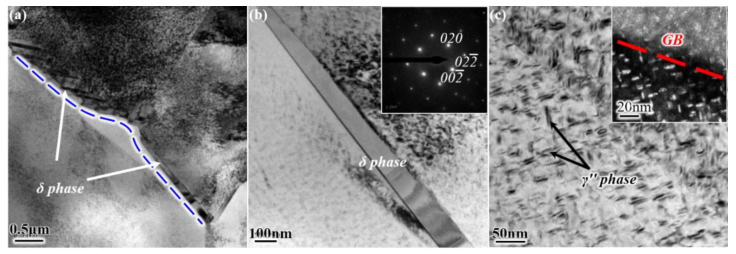
TEM observations revealing the δ phase and γ″ phase on TD–RD plane in (**a**,**b**) 940-TD sample and (**c**) 980-TD sample.

More details are revealed by TEM observations in Figure 4. Nano-sized precipitations of the γ″ phase are produced in grain interiors, whereas needle-like δ phases are precipitated on the grain boundary at 940 °C (Figure 4a,b). In parallel, a precipitation-free zone with a width of several hundreds of nanometers is formed in the vicinity of the grain boundary (highlighted by dotted line). As the temperature increases to 980 °C, the δ phase and the concomitant precipitation-free zone are invisible, and substantial γ″ phases are observed instead (Figure 4c).

**Figure 5:**
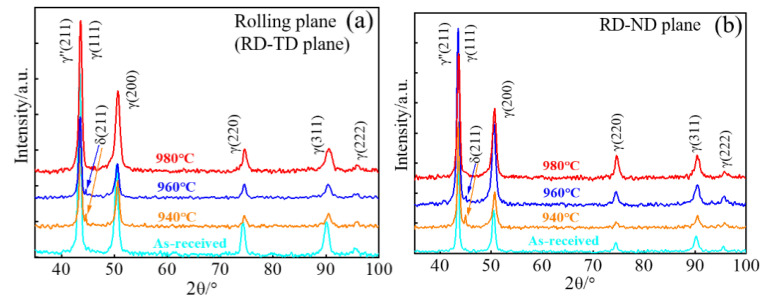
XRD patterns on (**a**) RD–TD and (**b**) RD–ND planes at various solid-solution temperatures.

Figure 5 shows the XRD profiles of the treated samples on rolling (RD–TD) and RD–ND planes. The matrix (γ phase) exhibits strong the (111) and (200) peaks with insignificant change before and after treatments on the rolling plane (Figure 5a). The solid-solution and aging treatments lead to the appearance of weak reflection peaks that correspond to the δ or γ″ phase. As the solid-solution temperature increases, the peak of the δ phase gradually disappears, while the peak of the γ″ phase is also observed. This indicates that aging treatment produces the γ″ phase, and the formation of the δ phase is attributed to the solid-solution treatment. For example, the gradual dissolution of the δ phase occurs after a solid-solution treatment at 960 °C, in accordance with SEM observations (Figure 2). Similar patterns are observed on the RD–ND plane, and there is no significant difference of XRD features, except the weak peak of the δ phase, among treated samples on both planes. This indicates that microstructures have not changed after various treatments and the orientation distribution of grains is not significantly different [13].

### 3.2. Room Temperature Tensile Properties

**Figure 6:**
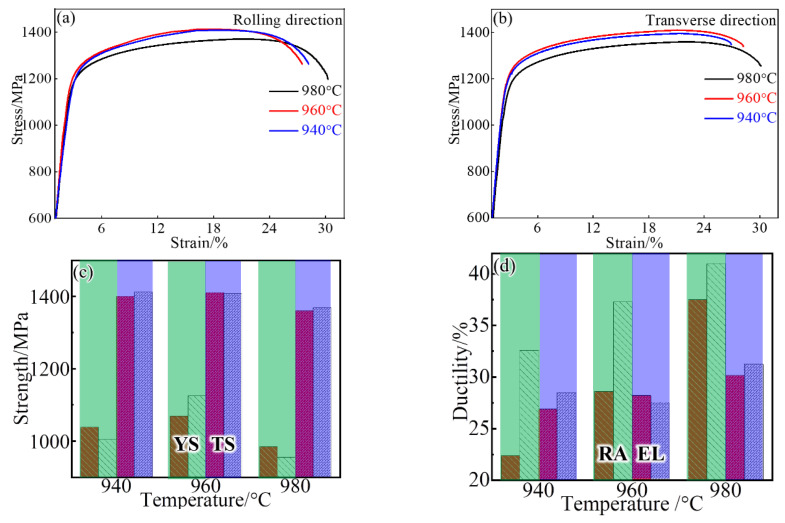
Typical stress–strain curves of the tensile samples tested at (**a**) RD and (**b**)TD, and their corresponding (**c**) strengths (YS and TS) and (**d**) ductile indexes (RA and EL) at different directions. The YS and RA are shown in the green frame of (**c**, **d**), respectively. The TS and EL are shown in the light purple frame of (**c**) and (**d**), respectively. Red bars represent the data at TD, whereas grey bars show the data at RD. (TS: tensile strength, YS: yield strength, EL: elongation, RA: reduction in area).

Figure 6 displays the stress–strain curves of samples that are treated at various temperatures and loaded along different directions (RD and TD). When loaded at RD, the sample after 940 °C treatment has a tensile strength (TS) of ~1400 MPa, and this strength does not increase at 960 °C but slightly decreases at a higher temperature of 980 °C (Figure 6a). In the 980 °C case, the increased ductility, including elongation (EL) and reduction in area (RA), is observed. The yield strength (YS) exhibits a similar trend, and its strength difference between samples is only ~100 MPa. When tested at TD, the tensile curves almost coincide with those of samples that are loaded at RD and treated at the same temperature. That is, there is no significant strength difference between TD and RD (Figure 6c). Moreover, the maximum strength is obtained at 960 °C, where the strengthening particles of the γ″ phase are precipitated at the expense of dissolution of the δ phase but without the significant grain coarsening (Figure 3). In contrast, the ductility, in particular for the RA, shows a strong dependence on the testing direction (Figure 6d). For example, the RA is ~22% and ~33% at TD and RD, respectively, for the sample after 940 °C treatment. As expected, an increased solid-solution temperature results in the improved RA, in particular at TD with a higher speed (highlighted by the red bar), and this causes the gradual decrease in the difference of the two directions.

**Figure 7:**
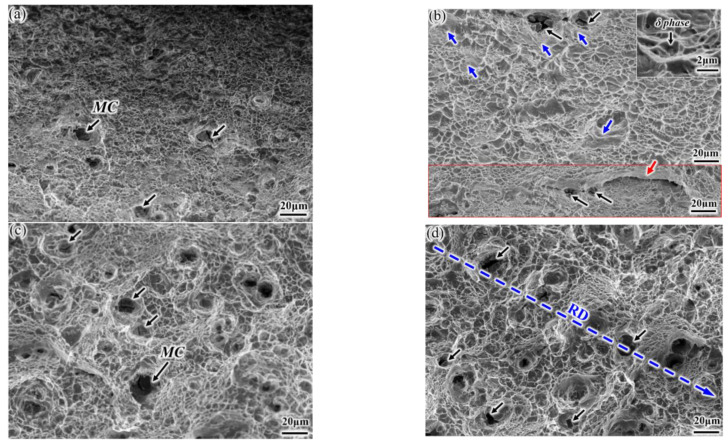
Fracture surfaces of tensile samples: (**a**) 940-RD, (**b**) 940-TD; (**c**) 980-RD; (**d**) 980-TD.

Typical fractured surfaces are examined under SEM, as shown in Figure 7. Ductile fractured feature of smaller dimples is observed on the surface of the 940-RD sample (Figure 7a), and there are still some big dimples with MC carbides located at the bottom (black arrow). Besides, δ phases also provide sites for dimple nucleation (Figure 7b), and the formed dimples are smaller than those associated with MC carbides. Bigger and shallower dimples supplemented by the brittle characteristics (blue arrow) are visible on the surface of the 940-TD sample. A typical example of the brittle intergranular feature is indicated by the red arrow, and this strip pattern corresponds to the almost continuous δ phase at the grain boundary (Figure 2a). This indicates that the 940-RD sample undergoes more deformation than the 940-TD sample. In contrast, larger and deeper dimples are observed on the surfaces of 980-TD and 980-RD samples, with the disappeared intergranular features. Besides, severe plastic tearing occurs. These aspects suggest that the elevated solid-solution temperature is conducive to ductility enhancement, in agreement with the tensile result (Figure 6). The huge dimples, which are nucleated at the micro-scale MC carbides, tend to be aligned along the RD (Figure 2c).

### 3.3. Room Temperature Impact Properties

**Figure 8:**
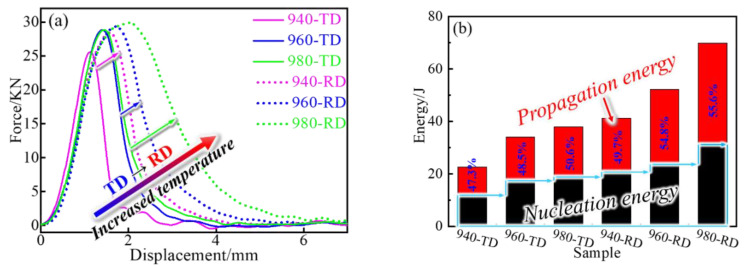
(**a**) Force–displacement curves of samples impacted at room temperature and (**b**) energy distribution for crack initiation and propagation.

The typical force–displacement curves for impact testing are shown in Figure 8a. Similar peak curves are observed, and the peak possesses the enlarged width and height at an increased treated temperature for both directions (RD and TD). The RD samples have a higher and broader peak than their TD counterparts, which indicates that more Charpy impact energy (CVN) is absorbed during testing for RD samples. For example, the energy value of the 940-RD sample (~41 J) is even bigger than that of the 980-TD sample (~38 J that also exceeds that of the 940-TD sample), as quantitatively shown in Figure 8b. That is, the strong anisotropy-dependent impact behavior (including crack initiation and propagation) is visible. It is easier for the crack to nucleate and propagate along the TD, in particular after the low-temperature treatment (i.e., the 940-TD sample). In contrast, the significantly increased resistance (i.e., two-fold increase for the 980-RD sample) emerges at the RD during both nucleation and propagation. Besides, less energy is consumed during crack propagation than during nucleation for the low-temperature treated samples or TD samples. Compared with the 940-TD sample, however, there is a negligible energy difference between nucleation and propagation after more increase in propagation energy for the 940-RD sample. This also reveals the anisotropic behavior, and the TD samples are more dangerous because sudden failure may occur during loading.

**Figure 9:**
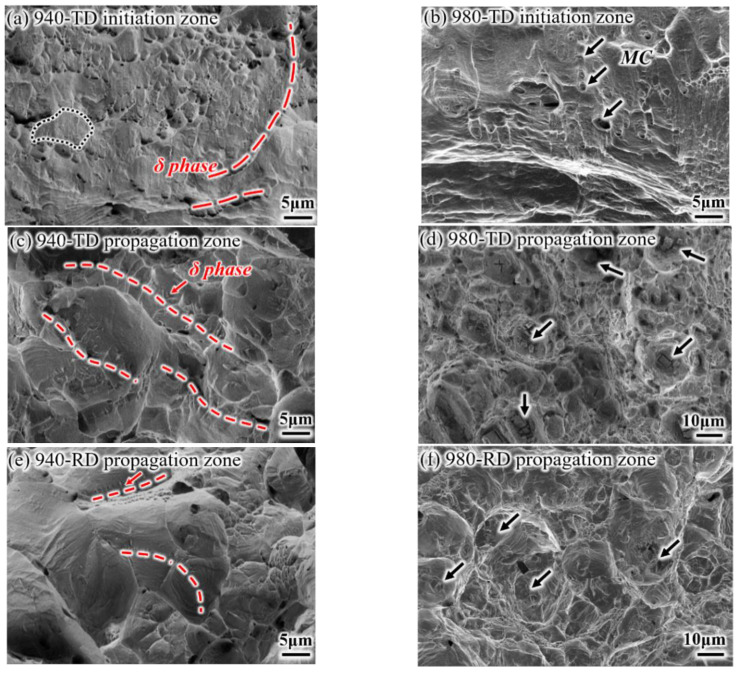
Representative fracture surfaces of impact samples: (**a**,**c**) 940-TD; (**b**,**d**) 980-TD; (**e**) 940-RD; (**f**) 980-RD.

**Figure 10:**
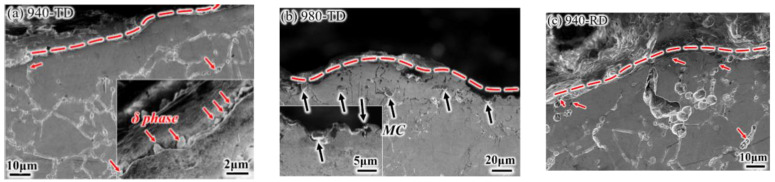
Profile fractograph of impact samples: (**a**) 940-TD; (**b**) 980-TD; (**c**) 940-RD.

Representative fractured surfaces and cross-sectional microstructures are shown in Figure 9 and Figure 10, respectively. A mixed morphology of brittle and ductile features, which consists of small dimples and intergranular patterns (i.e., grain is depicted by the black line), is observed at the crack initiation zone of the 940-TD sample (Figure 9a). Cracks nucleates at the grain boundaries distributed with δ phases, in particular for the string shape (highlighted by the red line). Its corresponding fracture profile (Figure 10a) demonstrates that the crack growth seems to occur along the grain boundary with δ phases and actually passes through the zone very adjacent to such grain boundary (insert). This produces small and shallow dimples on grain boundary facets that are also not smooth. The 980-TD sample also fails in a mixed mode with the main brittle characteristic (Figure 9b), which corresponds to the lower nucleation energy for TD samples (Figure 8b). Similar nucleation and growth behaviors, which associate with MC carbides instead of δ phases, are observed (Figure 10b), and these carbides are aligned nearly parallel to the loading direction (Figure 9b). Significant brittle features with smooth grain boundary facets are observed at the crack propagation zone of the 940-TD sample (Figure 9c), which is in agreement with the lower propagation energy than nucleation energy (Figure 8b). In contrast, the slightly increased nucleation energy is obtained in the 980-TD sample (Figure 8b), evidenced by the gradual appearance of plastic tearing at the propagation zone (Figure 9d). Compared with TD samples (940-TD and 980-TD), the increased ductile characteristics, e.g., the deformed grains and smaller dimples on grain boundary in Figure 9e and substantial dimples in Figure 9f, are observed in their RD counterparts. This is in accordance with the fact that RD samples consume more energy during crack propagation than their corresponding TD samples (Figure 8b).

### 3.4. Stress Rupture Properties of IN718 Alloys in High Temperature

**Figure 11:**
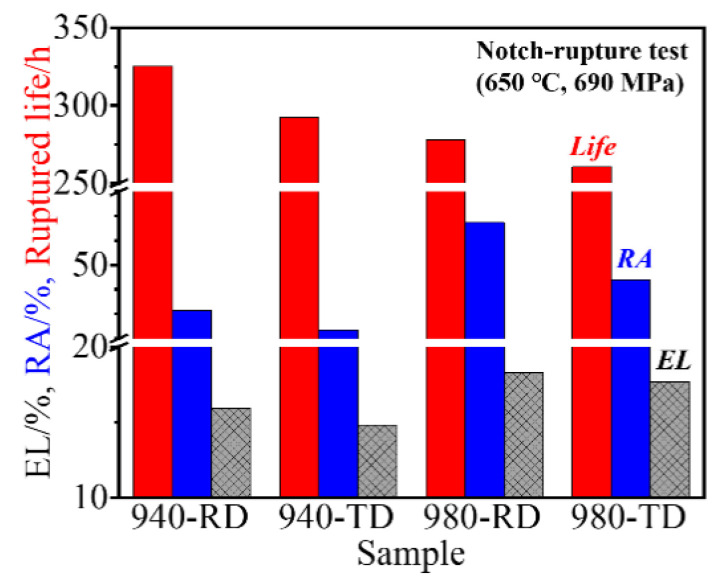
The rupture properties of samples tested at 650 °C and 690 MPa.

**Figure 12:**
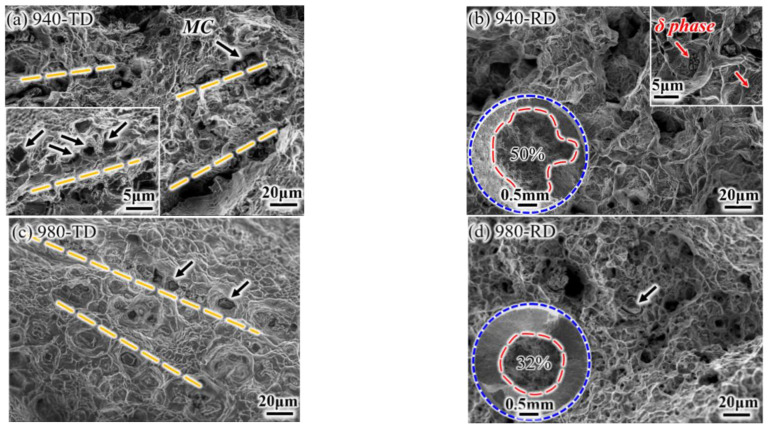
Representative fracture surfaces of rupture samples: (**a**) 940-TD; (**b**) 940-RD; (**c**) 980-TD; (**d**) 980-RD.

**Figure 13:**
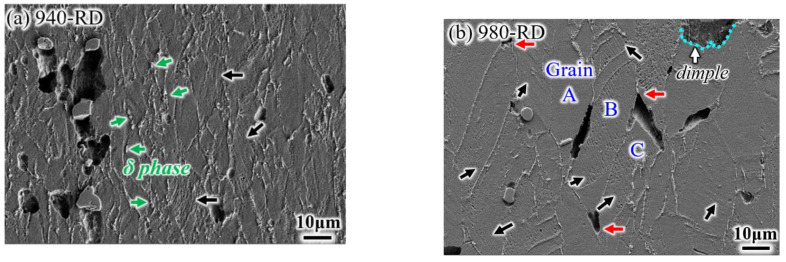
Profile fractograph of rupture samples: (**a**) 940-RD; (**b**) 980-RD.

The stress rupture test was performed at 650 °C with the applied stress of 690 MPa. All samples fractured at the smooth gauge part rather than at the notch, which indicates the insignificant notch sensitivity at the elevated temperature. The RD samples have a longer rupture life than TD samples, and such life is also more at the lower treated temperature (Figure 11). For example, it takes more time to rupture for the 940-TD sample than for the 980-RD sample. This indicates that the treated temperature has more impact on the ruptured life than the loading direction. The rupture ductility (including EL and RA) is slightly improved at the RD or at the higher treated temperature. In fact, there is not substantial ductility difference between loading directions or solid-solution temperatures when tested at the elevated temperature of 650 °C, in comparison with the significant ductility difference at room temperature (Figure 6d). A dimpled fracture occurred in the 940-TD sample without the significant intergranular characteristics (Figure 12a), which were observed on the corresponding tensile fracture surface tested at room temperature (Figure 7a). Cracks are visible along grain boundary, which is produced during the up-link of microvoids nucleating at MC carbides with the linear alignment. The formation of dimples is also ascribed to δ phases, as representatively shown in the insert of Figure 12b. Moreover, the transgranular fracture occurred in the 940-TD sample, whereas the shape of grains is faintly visible on the fractured surface (Figure 12b). Typical dimpled surfaces are observed in 980-TD and 980-RD samples, which indicates the increased ductility, as also evidenced by the smaller fibrous zone (inserts of Figure 12b,d). Dimples containing the MC carbides tend to align in a row when loaded at TD, i.e., for both 980-TD and 940-TD samples (Figure 12a,c). Dimples seem to be linked with a segment of grain boundary, as shown in fracture profiles in Figure 13. Therefore, these dimples are not the same as those produced at room temperature (Figure 7c,d). Significant slip characteristics are observed in 980-RD and 940-RD samples (highlighted by the black arrow in Figure 13), and the bent slip lines indicate that the whole grain undergoes the deformation. Cracking occurs at the grain boundary where there is a marked difference between slip systems in adjacent grains, i.e., grain A and B (Figure 13b). The triple junctions and the large MC carbides also serve as the preferential sites for crack nucleation (red arrow), in particular in the coarse-grained sample, i.e., 980-RD (Figure 13b). In contrast, no cracks nucleated at the small δ phases are detected in fine-grained sample 940-TD sample (Figure 13a).

## 4. Discussion

**Figure 14:**
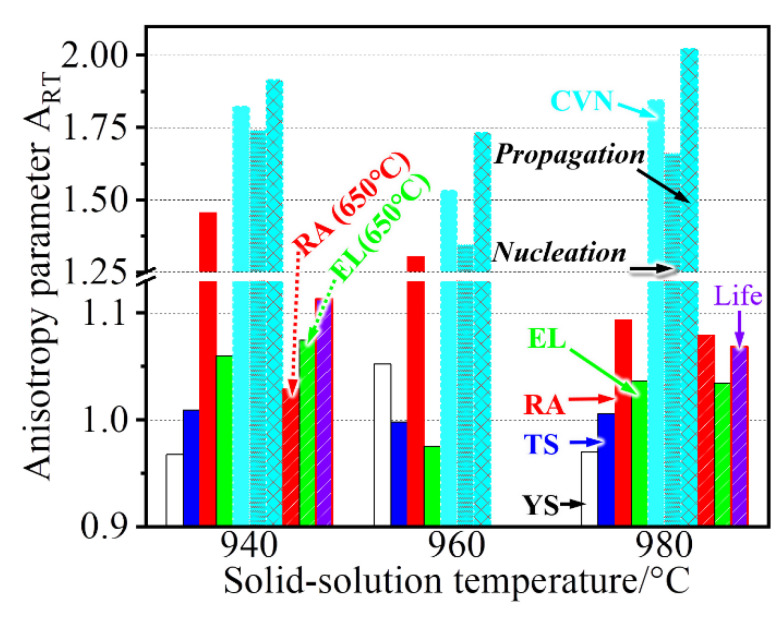
Anisotropy parameter comparison of samples treated at different temperatures.

In this work, significant differences in room temperature ductility (in particular for RA) and toughness (CVN) were observed at two testing directions or in two sample orientations (Figure 6 and Figure 8). These properties present a sound anisotropy behavior that is dependent on the direction or orientation, whereas a weak anisotropy in the strengths (TS and YS) and rupture properties at 650 °C is visible. For evaluation, the anisotropy parameter *A_RT_* can be quantitatively determined by [11]:

(1) $$A_{RT}=\frac{P_{RD}}{P_{TD}}$$

where *P_RD_* and *P_TD_* are the corresponding mechanical properties tested at *RD* and *TD*, respectively. The *A_RT_* value getting closer to 1 indicates that such property tends to be more isotropic, whereas its deviation exceeding 0.1 (i.e., 1.1 or 0.9) suggests the existence of anisotropy. In this case, RD or TD performance is 10% larger than its counterpart. The anisotropy parameters of various properties are given in Figure 14. A higher *A_RT_* value of ~1.75 is obtained for impact properties, and such value is further increased to ~2 at 980 °C. In contrast, an increased solid-solution temperature leads to a decreased *A_RT_* value for the RA. This evidence implies that the treated temperature also has some impact on the anisotropy. Another significant phenomenon is the decreased anisotropy for the high-temperature ductility even at 940 °C, which greatly contrasts with room-temperature ductility. This indicates that different deformation behaviors at two tested temperatures may have an effect on the anisotropy, which will be discussed.

The crystallographic texture is frequently reported to produce significantly anisotropic properties, i.e., the YS [14] and Charpy toughness [15] in the pipeline steels. The pronounced texture discrepancy is undetected, evidenced by the similar XRD patterns on the RD–TD and RD–ND planes even after various treatments (Figure 5). The present treatments result in a relatively uniform microstructure (Figure 2) rather than the deformation-induced lamellar microstructure that yields an orientation dependent strength [16], and there is only a minor size difference among grains (Figure 3). Moreover, there are well-distributed γ′/γ″ precipitations that do not align on the {111} habit/slipping planes, where the anisotropy of strength is reported to be enhanced by Ω phase in the Al alloy [17]. Actually, such coherent precipitations may cause the anisotropic behavior in flow stress but with the obligatory presence of crystallographic texture [18]. In contrast, the non-sharable particles, i.e., MC carbides, promote the operation of slip systems [19] and thus are conducive to weakening the anisotropy [20]. Although the plate-shaped δ phases seem to be aligned along the RD, the arbitrary slip systems are likely to be activated in neighboring grains that are randomly distributed. These aspects account for the weak anisotropy of YS in Figure 14.

**Figure 15:**
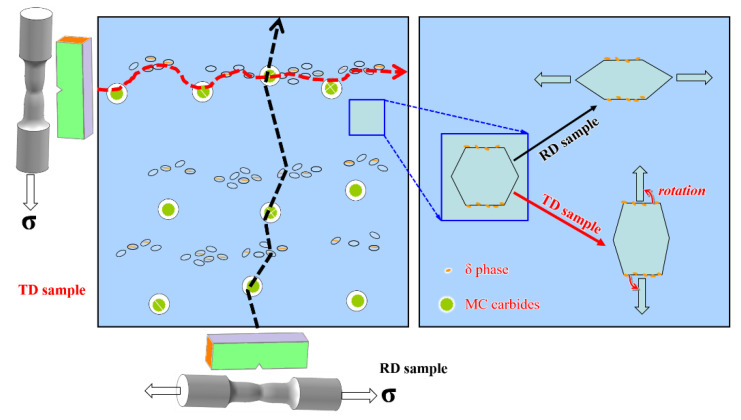
A schematic showing the crack nucleation and propagation at RD and TD.

The YS anisotropy arises from the difficulty degree when operating slip systems at various loading directions [21], whereas the ductility/toughness anisotropy is dependent on the subsequent deformation and fracture behaviors of materials, including crack initiation and propagation. After yielding, the increased loading leads to uniform deformation, particularly with the help of particles that play a role in slip homogenization [22]. In this case, micro-voids are initiated continually by the debonding or breaking of large particles, i.e., MC carbides, but without propagation because of the excellent fracture toughness for the face-centered cubic matrix. Until necking that corresponds to the TS, nonuniform and localized deformation starts, followed by void coalescence and linkage. The particles have a minor impact on TS and EL that mainly consists of uniform strain, and thus their *A_RT_* values are only ~1. The RA, however, is an indicator of localized strain at the necking position during coalescence and linkage of particle-induced voids, and strong anisotropy is observed. For example, high-density particles trigger the formation of voids that are closer to each another, as shown in Figure 15, and this results in a more rapid coalescence and failure process in TD sample than that in RD sample. Substantial dissolution of δ phases at a higher solid-solution temperature (i.e., 980 °C) reduces the initiation sites of voids but widens their bridged distance. As a result, an indistinct difference in coalescence behavior is gradually formed between RD and TD, evidenced by the decreased *A_RT_* value at the rising treated temperature (Figure 14). Compared with quasi-static deformation (tensile testing), dynamic deformation (impact testing) is more sensitive to these particles. It is easier for the crack to nucleate and propagate when more particles participate in the dense formation and rapid growth of micro-voids, and thus less energy is consumed during both nucleation and propagation, i.e., 940-TD sample (Figure 8). The increased nucleation energy and propagation energy are obtained through approaches that lower the particle density, i.e., dissolution of δ phases at elevated temperatures (960-TD and 980-TD samples) and an alteration of loading direction (940-RD sample). Therefore, the *A_RT_* values maintain a high degree (~1.75) at 940 °C treatment (Figure 14) and are decreased, particularly for the value of propagation energy, after the 960 °C treatment that produces the low-density particles. Although δ phases are almost dissolved at 980 °C, a small amount of the unaltered MC carbides aligned along the RD cause the stress concentration in the enlarged grains that largely stimulates the formation and coalescence of voids. This results in increased *A_RT_* values, particularly for the value of propagation energy exceeding 2.0 and also larger than that at 940 °C, because the refined grains facilitate the delaying of large-scale localized deformation [19]. Besides, its *A_RT_* value of nucleation energy at 980 °C is close to that at 940 °C, which indicates that coarsened grains have a similar impact as δ phases on the anisotropy in this work.

The grain boundary particle always results in the stress concentration during room-temperature loading irrespective of its morphologies. In contrast, the granular δ phase possesses good deformation compatibility with grain boundaries and can hinder crack growth via the accommodation of crack path in a transgranular manner at elevated temperature, i.e., 650 °C [23]. That is, such particles show two opposite sides during the void coalescence process (corresponding to the localized deformation): acceleration at room temperature and deceleration at elevated temperature. Therefore, RA is greatly improved in the fine-grained 940 °C samples that have the nearly granular δ precipitations. For example, the RA of the 940-TD sample doubles, from ~22% tested at room temperature (Figure 6d) to ~46% tested at 650 °C (Figure 11). This largely counteracts the competitive effects of testing directions and leads to the significant reduction of *A_RT_* value for RA at 650 °C (Figure 14). Moreover, δ precipitations impede dislocation movements [24], particularly for the needle-like ones (Figure 4a,b) or high-density ones (i.e., the nearly continuous δ phase at local grain boundaries in Figure 2a). Such substantial δ phases may cause stress concentration near themselves and microcrack nucleation during the later stage [8]. This is synergized by the micro-plastic zone that depletes the strengthening γ″-Ni3Nb phase around δ-Ni3Nb phase and is ductile but significantly weak in strength. After some deformation, failure preferentially occurs at this micro-zone, and thus grain boundaries with some plastic tearing are faintly observed in Figure 12a,b.

In the case of elevated temperatures, the strength degradation takes place in both grain interior and grain boundary, particularly for the latter one. Deformation occurs not only via the dislocation movements but through the operative grain boundary processes and diffusional creep. As illustratively shown in Figure 13b, grains are subjected to a morphological transformation from equiaxed to elongated after rupture testing, which confirms the emergence of grain boundary sliding. This is also evidenced by the formation of voids at triple points where there is stress concentration during grain boundary sliding [25]. However, grain boundary motions are strongly pinned by undissolved δ phases in the refine-grained 940 °C samples (Figure 13a), and such pinning largely compensates for the loss of grain boundary strength. Notably, both necking (void coalescence) and crack propagation are effectively delayed by globular δ phases [23]. The rupture process is thus slowed down, and the rupture life is more prolonged in fine-grained samples (940-RD and 940-TD) than that in coarse-grained samples (980-RD and 980-TD). In the case of coarse-grained samples without δ phases, a wider linkage distance between voids nucleated at the stringed MC carbides (Figure 13b) should be mainly responsible for the slightly longer life in 980-RD sample, whereas insignificant anisotropy is observed due to the countable carbides. Its *A_RT_* value of ~1.07 is underestimated, because 980-RD sample undergoes more time (~17.7 h) at a higher stress (759–828 MPa). Only ~25% of rupture life (~58 h) is retained in double-aged IN718 alloy when the tested stress increases from 625 MPa to 700 MPa at 650 °C [26]. Therefore, the nearly linear-aligned carbides, even with a limited amount, have a gradually increased role in the anisotropic behavior of rupture life, particularly when the string of δ phases also coexists, i.e., in fine-grained samples. On the one hand, it is quite difficult for the grain boundary pinned by δ phases to rotate from RD to the loading direction TD with a bigger rotation angle (Figure 15). Conversely, deformation, i.e., shrinking, occurs facilely at TD for the grain boundary without δ phases, and elongated grains are developed when tested at RD (Figure 13a). On the other hand, substantial initiation of voids, which is cooperatively induced by carbides and δ phases, largely shortens the linkage distance during void coalescence. As a result, a relatively strong anisotropy is expected to be observed in fine-grained 940 °C samples, and its *A_RT_* value should be much larger than the measured value of ~1.1 in Figure 14. This is attributed to a similar fact that ignores the contribution of the extra loading at a significantly increased stress of 897-1035 MPa for a longer duration of 33 h. Furthermore, dislocation motions are also responsible for the creep properties, evidenced by substantial creep voids nucleated at the dense slip bands in the grain interior, particularly for the coarse-grained samples (Figure 13). This partly accounts for the shorter creep life in coarse-grained samples that undergo more deformation than fine-grained samples in both matrix and grain boundaries, which corresponds to the uniform and nonuniform deformation, respectively. This, in turn, leads to the enhanced EL and RA at 650 °C (Figure 11).

## 5. Conclusions

In this present work, microstructure and mechanical anisotropy are comprehensively investigated in a slightly rolled Inconel718 sheet that suffers from solid-solution and aging treatments. The results are summarized as follows:

(1) A weak anisotropy in the strengths is attributed to the MC carbides that promote the operation of slip systems during room temperature tension;
(2) The strong anisotropy in RA is mainly affected by string-like δ phases that provide more sites during crack nucleation but also stimulate rapid linkage during propagation;
(3) Both MC carbides and δ phases are responsible for the strong anisotropy in CVN at a solid-solution temperature of 940 °C, whereas MC carbides and grain coarsening account for the sound anisotropy in CVN at 980 °C where δ phases are dissolved;
(4) The grain boundary processes are operated at the elevated temperature of 650 °C, leading to a weak anisotropy of rupture properties. Strong pinning of δ phases causes a longer rupture life in the refine-grained 940 °C samples than that in coarse-grained 980 °C samples.

## Data Availability

Not applicable.
